# Environment-Dependent UV Response of Cyclosporin A in Buffered Aqueous Media: Implications for Reliable Quantitative Analysis in Ophthalmic Drug-Delivery Research

**DOI:** 10.3390/pharmaceutics18080956

**Published:** 2026-08-03

**Authors:** Iwona Nowak, Ola Michałkiewicz, Iwona Rykowska, Rafał Nowak

**Affiliations:** 1 Faculty of Chemistry, Adam Mickiewicz University, Uniwersytetu Poznańskiego 8, 61-614 Poznań, Poland; ola.michalkiewicz@amu.edu.pl (O.M.);; 2 Department of Ophthalmology, Józef Struś City Hospital, Szwajcarska 3, 61-285 Poznań, Poland

**Keywords:** cyclosporin A, UV–Vis spectroscopy, pH-dependent spectral response, ocular drug delivery, contact lenses, artificial lacrimal media, analytical quantification, principal component analysis, Gaussian deconvolution

## Abstract

**Background:** Cyclosporin A (CyA) is widely investigated for ophthalmic drug-delivery systems, including hydrogel contact lenses, where reliable quantification is essential for evaluating drug loading, release, and formulation performance. However, the analytical UV response of CyA may depend on the physicochemical characteristics of the surrounding buffered environment, potentially affecting quantitative measurements. **Objectives:** This study aimed to determine whether changes in the buffered aqueous environment influence the UV spectral response of CyA and whether such variability may introduce systematic bias into UV-based quantitative analysis. **Methods:** UV spectra of CyA were recorded in citrate-phosphate buffered media over the pH range 3.28–9.10, including several near-neutral conditions relevant to ophthalmic formulations and artificial lacrimal media. Spectral changes were evaluated using univariate statistical analysis, principal component analysis (PCA), and Gaussian deconvolution. **Results:** CyA exhibited a progressive hypsochromic shift of the apparent absorption maximum from approximately 234 nm to 210–214 nm, accompanied by pronounced changes in absorbance intensity and the overall absorption profile. Comprehensive statistical analyses demonstrated systematic spectral differences across the investigated buffered media. PCA performed on 42 spectra (31 wavelength variables) explained 84.20% of the total variance within the first two principal components, while Gaussian deconvolution indicated progressive redistribution of overlapping spectral contributions. The observed spectral behavior is consistent with changes in the molecular environment and redistribution of conformational populations; however, the UV–Vis data alone do not provide direct structural evidence for these processes. The resulting wavelength-dependent spectral variability produced substantial apparent analytical bias, demonstrating that fixed-wavelength UV measurements may lead to systematic quantification errors when calibration standards and analytical samples differ in their buffered environment. **Conclusions:** These findings demonstrate that the analytical UV response of cyclosporin A is environment-dependent and highlight the importance of matrix-matched calibration and validation under the intended analytical conditions when UV spectroscopy is used for quantitative determination of CyA in aqueous drug-delivery systems.

## 1. Introduction

Cyclosporin A (CyA) is a cyclic undecapeptide widely used as an immunosuppressive agent in ophthalmology, particularly in the treatment of dry eye disease and inflammatory ocular conditions. In recent years, CyA has also emerged as an important model compound in advanced ocular drug-delivery systems, including hydrogel-based contact lenses designed for sustained drug release [1,2,3,4,5]. In this context, biopolymer- and hydrogel-based ocular drug-delivery systems provide particularly relevant matrices, as their hydrated polymeric structure may influence drug solvation, retention, and release behavior [6].

Despite its clinical relevance, the physicochemical behavior of CyA in aqueous environments remains analytically challenging. Due to its hydrophobic character and macrocyclic structure, CyA exhibits limited aqueous solubility together with a complex conformational landscape [7,8,9,10]. A network of intramolecular hydrogen bonds stabilizes its structure, while interactions with the surrounding solvent significantly influence its conformational ensemble [7,8,9,10,11].

UV–Vis spectrophotometry is commonly employed for the quantitative determination of CyA, typically using wavelengths around 210–214 nm corresponding to peptide π→π* transitions [12,13]. However, peptide chromophores exhibit broad and overlapping absorption bands that are highly sensitive to solvation, hydrogen bonding, and conformational state. Accordingly, the assumption of a constant molar absorptivity at a fixed wavelength may not be valid when calibration standards and analytical samples differ in their physicochemical environment, potentially introducing systematic errors into UV-based quantification.

Recent studies have demonstrated that CyA displays pronounced conformational flexibility and environment-dependent behavior, including solvent-induced reorganization and cis/trans isomerization of amide bonds [7,8,9,10,11]. However, the influence of pH on its UV spectral response and the resulting implications for analytical quantification have not been, to the best of our knowledge, systematically investigated.

This represents a critical gap, particularly in the context of aqueous drug-delivery systems. In hydrogel-based formulations, such as therapeutic contact lenses, the local microenvironment may differ significantly from bulk solution conditions due to polymer–drug interactions, hydration gradients, and spatial pH variations.

For ocular applications, the physicochemical characteristics of the analytical medium are not merely technical variables. Among these, pH represents one of the most relevant formulation parameters. Commercial cyclosporine ophthalmic products are designed for topical administration to the ocular surface. For example, RESTASIS^®^ (cyclosporine ophthalmic emulsion 0.05%) is indicated to increase tear production in patients with inflammation-associated suppression of lacrimal secretion in keratoconjunctivitis sicca. Its formulation is reported to have a pH of 6.5–8.0 [14]. Other commercially available ophthalmic cyclosporin A formulations further illustrate the diversity of formulation approaches. CEQUA^®^ is available as a nanomicellar aqueous solution, whereas Ikervis^®^ is formulated as a cationic emulsion designed to prolong ocular surface residence time.

This pH interval provides a clinically and formulation-relevant reference range for interpreting the analytical behavior of cyclosporin A under near-neutral conditions. Accordingly, UV-based measurements performed in this pH region are directly relevant to ocular formulation development and to in vitro release studies conducted in artificial lacrimal or buffer-based media.

Therefore, this study aimed to evaluate the pH-dependent analytical spectral response of cyclosporin A with particular emphasis on its implications for UV-based quantification in ophthalmic drug-delivery research. Rather than assuming that the UV spectrum of CyA represents an invariant analytical fingerprint, we investigated whether changes in pH modify its analytical spectral response, including the apparent absorption maximum, band profile, and wavelength-dependent absorbance in aqueous media.

Based on these considerations, we hypothesized that pH-dependent changes in the analytical spectral response of CyA may introduce systematic quantification bias when calibration standards and analytical samples differ in their physicochemical environment.

## 2. Materials and Methods

### 2.1. Preparation of CyA Solutions

Cyclosporin A (TCI, Europe) solutions were prepared at a concentration of 20 µg/mL, corresponding to approximately 16.6 µM. For each pH condition, three CyA solutions were prepared independently by adding CyA separately to the corresponding buffer medium, resulting in three independent experimental replicates. The CyA stock solution was maintained at 4 °C in a thermoshaker until immediately before addition to the buffer. All analytical solutions remained visually clear throughout sample preparation and measurement, with no visible precipitation or turbidity. This experimental design was adopted to eliminate the influence of organic solvents and to evaluate the analytical UV response of CyA exclusively in buffered aqueous media.

The citrate–phosphate buffer components were maintained at room temperature. After addition of CyA to the corresponding buffer directly in a quartz cuvette, the solution was mixed and the spectrum was recorded immediately, followed by two additional recordings at approximately 30 s intervals. No changes in either the position or the shape of the absorption band were observed during this period; therefore, the final spectrum was used for subsequent analysis.

The possible contribution of aggregation or self-association to the observed analytical spectral response was not investigated in the present study.

A citrate–phosphate buffer system was used to obtain the pH range 3.28–9.10. The pH of the solutions was controlled by adjusting the relative volumes of the two buffer components. All buffers were prepared to a final volume of 25 mL by mixing the volumes of 0.1 M Na_2_HPO_4_ and 0.1 M citric acid specified in Table 1. The composition of the buffer solutions is summarized in Table 1.

The investigated pH series included several points in the near-neutral interval (pH 6.60, 6.75, 6.99, 7.37, 7.70, and 7.99), enabling targeted interpretation of the spectral response within a range relevant to ophthalmic formulation and release media.

### 2.2. UV–Vis Spectroscopy

UV spectra were recorded in the 190–800 nm range using quartz cuvettes. For detailed analysis, the spectral range of 200 to 300 nm was selected to cover the far-UV region, where absorption bands associated with peptide amide chromophores are expected [12,13].

For each pH condition, UV spectra were recorded for three independently prepared replicate solutions. Mean spectra were subsequently used for descriptive spectral analysis, whereas all statistical analyses were performed using individual replicate measurements.

Baseline correction was applied to eliminate solvent contributions. For descriptive visualization, spectra were evaluated in both raw and normalized forms to facilitate comparison of band shape. Statistical analyses and chemometric calculations were performed using baseline-corrected spectra without normalization unless stated otherwise.

The experimental conditions are summarized in Table 2.

### 2.3. Analytical Evaluation

The analytical response of CyA was evaluated using absorbance values measured at two commonly employed analytical wavelengths (206 and 234 nm). Apparent relative deviation was subsequently calculated to estimate the potential bias that would arise if calibration established under one set of conditions were applied to samples measured under different buffer conditions.

The apparent relative deviation was calculated according to:

$$Apparent\;relative\;deviation\;(\%)=\left[\frac{\left(A_{\lambda }-A_{ref}\right)}{A_{ref}}\right]\times 100$$

where $A_{\lambda }$ is the absorbance measured at the selected analytical wavelength and $A_{ref}$ is the absorbance used as the reference signal under matched experimental conditions. These values should be interpreted as apparent analytical quantification bias rather than full method-validation errors. This approach enabled quantification of the analytical bias associated with wavelength selection.

The objective of this analysis was not to validate a universal quantitative method but to evaluate the transferability of UV-based analytical response across buffered media differing in physicochemical composition.

### 2.4. Statistical Analysis

Statistical analyses were performed using three independently prepared replicate solutions for each pH condition (n=3). Descriptive data are presented as mean ± standard deviation (SD). Spectral descriptors were calculated separately for each replicate and included the apparent absorption maximum ($\lambda_{max}$), absorbance at $\lambda_{max}$ absorbance at 206, 224, and 234 nm, integrated spectral area over 200–260 nm, and spectral centroid.

Differences among pH conditions were evaluated using one-way analysis of variance (ANOVA). Homogeneity of variance was assessed using Levene’s test based on the median. Tukey’s honestly significant difference (HSD) test was used for multiplicity-adjusted pairwise comparisons. Because only three independent observations were available for each pH condition, group-wise normality tests were not considered sufficiently informative. Kruskal–Wallis tests were therefore additionally performed as non-parametric sensitivity analyses.

The apparent $\lambda_{max}$ values were recorded at discrete 2 nm intervals and frequently showed no within-group variability. Consequently, $\lambda_{max}$ was analyzed using the Kruskal–Wallis test, followed by pairwise Mann–Whitney U tests with Holm adjustment for multiple comparisons. Effect sizes were reported as eta-squared (η^2^) and omega-squared (ω^2^) for ANOVA and epsilon-squared (ε^2^) for Kruskal–Wallis tests. Statistical significance was defined as *p* < 0.05.

All analyses were performed using Python 3.13.5 with SciPy and statsmodels.

### 2.5. Chemometric Analysis

#### 2.5.1. Principal Component Analysis (PCA)

Principal component analysis (PCA) was applied as an exploratory multivariate technique to evaluate and visualize differences in the analytical spectral response of CyA across the investigated buffered media [15]. The analysis was performed using individual UV spectra acquired from three independently prepared replicate solutions for each pH condition. The data matrix comprised 42 individual spectra and absorbance variables spanning the 200–260 nm wavelength region. Prior to PCA, spectra were baseline-corrected and mean-centered without additional scaling, thereby preserving the relative contribution of wavelength regions exhibiting the greatest analytical variance. No spectral normalization was applied.

The number of principal components retained for interpretation was determined from the cumulative explained variance and inspection of score and loading plots. Score plots were subsequently used to visualize clustering of spectra according to pH, whereas loading plots were examined to identify wavelength regions contributing most strongly to the observed spectral variation.

Multivariate differences among pH groups were additionally evaluated using permutational multivariate analysis of variance (PERMANOVA), whereas homogeneity of multivariate dispersion was assessed using PERMDISP.

#### 2.5.2. Spectral Deconvolution

The absorption band (200–260 nm) was fitted using a two-component Gaussian model. Nonlinear least-squares fitting was applied to determine peak position, width, and relative contribution.

Goodness of fit was evaluated using residual analysis and the coefficient of determination ($R^{2}$). Gaussian deconvolution was applied as a mathematical tool to describe the evolution of overlapping spectral components. The resulting component bands were interpreted as phenomenological mathematical descriptors of the experimental spectra and were not assigned to individual electronic transitions or specific molecular conformations.

## 3. Results

### 3.1. pH-Dependent Analytical Spectral Response of CyA

**Figure 1:**
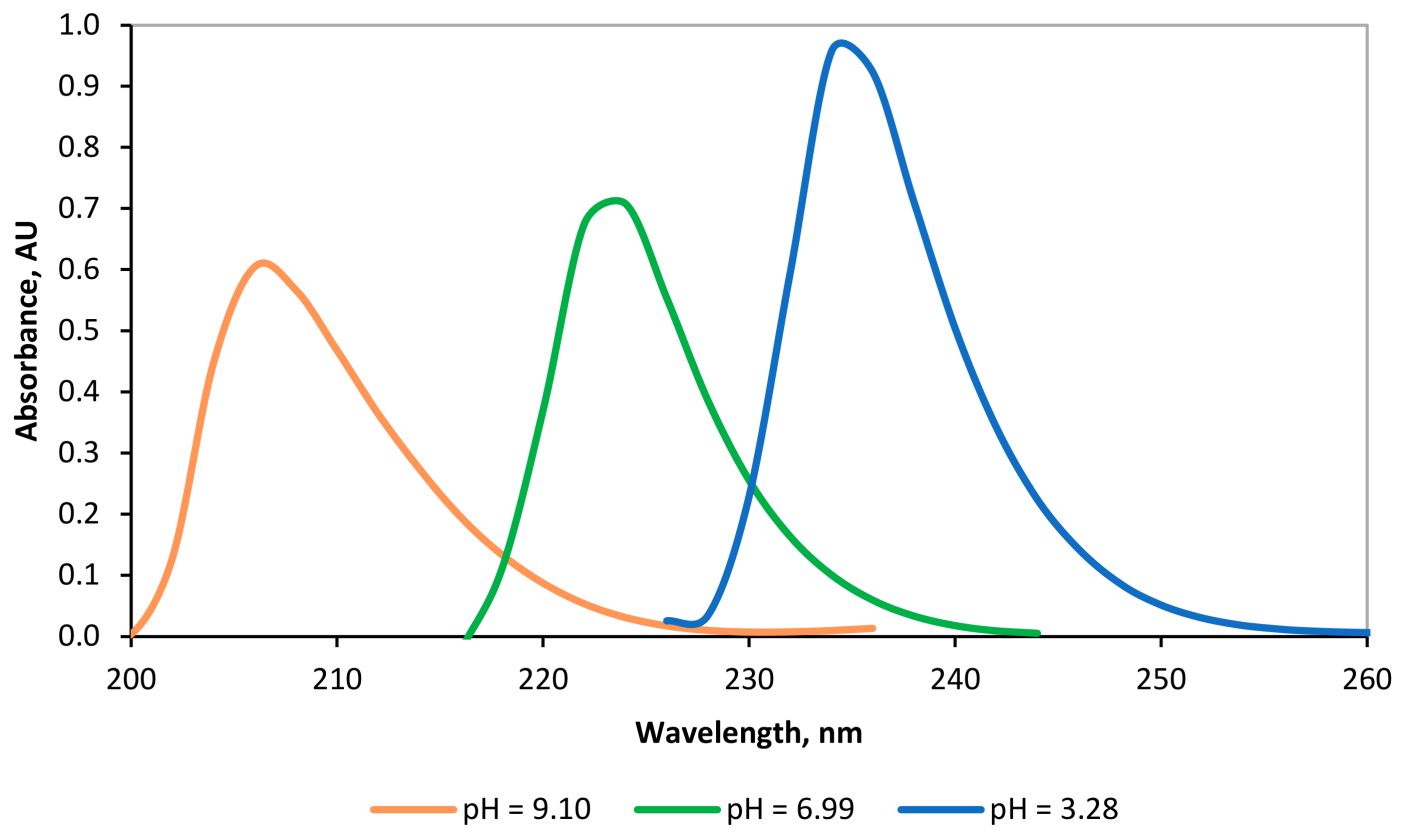
UV absorption spectra of cyclosporin A recorded in citrate–phosphate buffered media of different pH, demonstrating a progressive hypsochromic shift accompanied by changes in absorbance intensity and band shape.

The UV spectra of CyA exhibited a pronounced pH-dependent analytical spectral response, characterized by a progressive hypsochromic shift in the apparent absorption maximum ($\lambda_{max}$) from approximately 234 nm under acidic conditions to 206–214 nm under alkaline conditions (Figure 1). The spectral shift was accompanied by a decrease in absorbance intensity and a marked modification of the overall absorption profile and band symmetry.

Quantitative analysis confirmed significant differences among the investigated buffered media for all evaluated spectral descriptors (Table 3). Across the full pH range, statistically significant differences were observed for the apparent absorption maximum ($\lambda_{max}$), absorbance at $\lambda_{max}$, absorbance at 206, 224, and 234 nm, integrated spectral area (200–260 nm), and spectral centroid (all *p* < 0.001). For the continuous descriptors analyzed by ANOVA, effect sizes were exceptionally large (η^2^ = 0.945–0.9999), indicating that differences among the buffered media accounted for most of the observed variability.

Statistically significant differences also remained evident within the near-neutral pH interval (6.60–7.99), demonstrating measurable variation in the analytical spectral response of CyA under conditions relevant to ophthalmic formulations and artificial lacrimal media (Table 3).

#### Spectral Response Under Strongly Acidic and Strongly Alkaline Conditions

**Figure 2:**
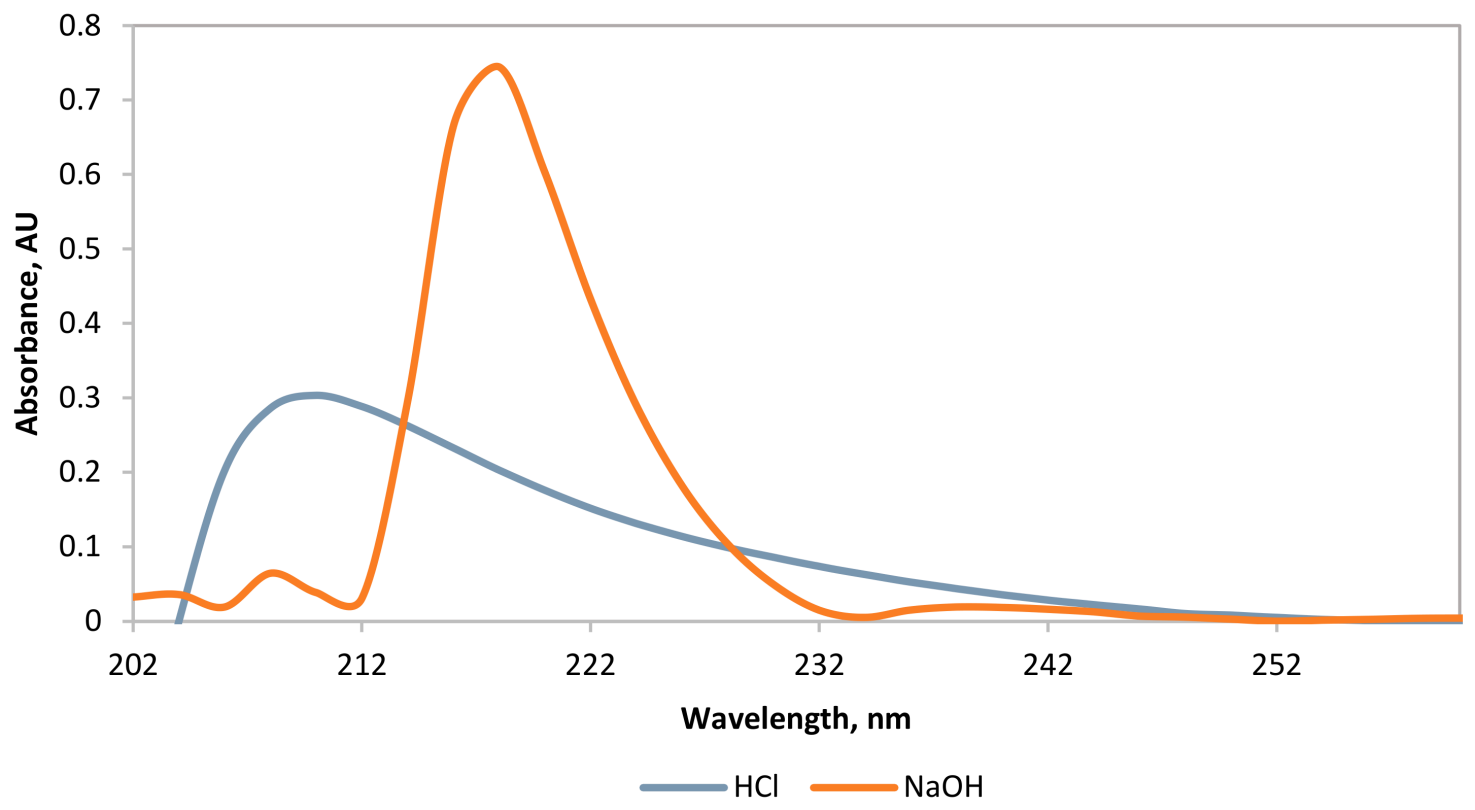
UV–Vis spectral response of cyclosporin A under strongly acidic (HCl) and strongly alkaline (NaOH) conditions.

The UV spectra recorded under strongly acidic (HCl) and strongly alkaline (NaOH) conditions exhibited pronounced differences in both the apparent absorption maximum and the overall absorption profile (Figure 2). Under alkaline conditions, the absorption band was narrower and more symmetric, whereas under acidic conditions a broader and more asymmetric profile was observed. Depending on the medium, the apparent absorption maximum occurred within the approximate range of 210–218 nm. No isosbestic point was observed in the investigated spectral region.

### 3.2. Principal Component Analysis of the pH-Dependent Analytical Spectral Response

**Figure 3:**
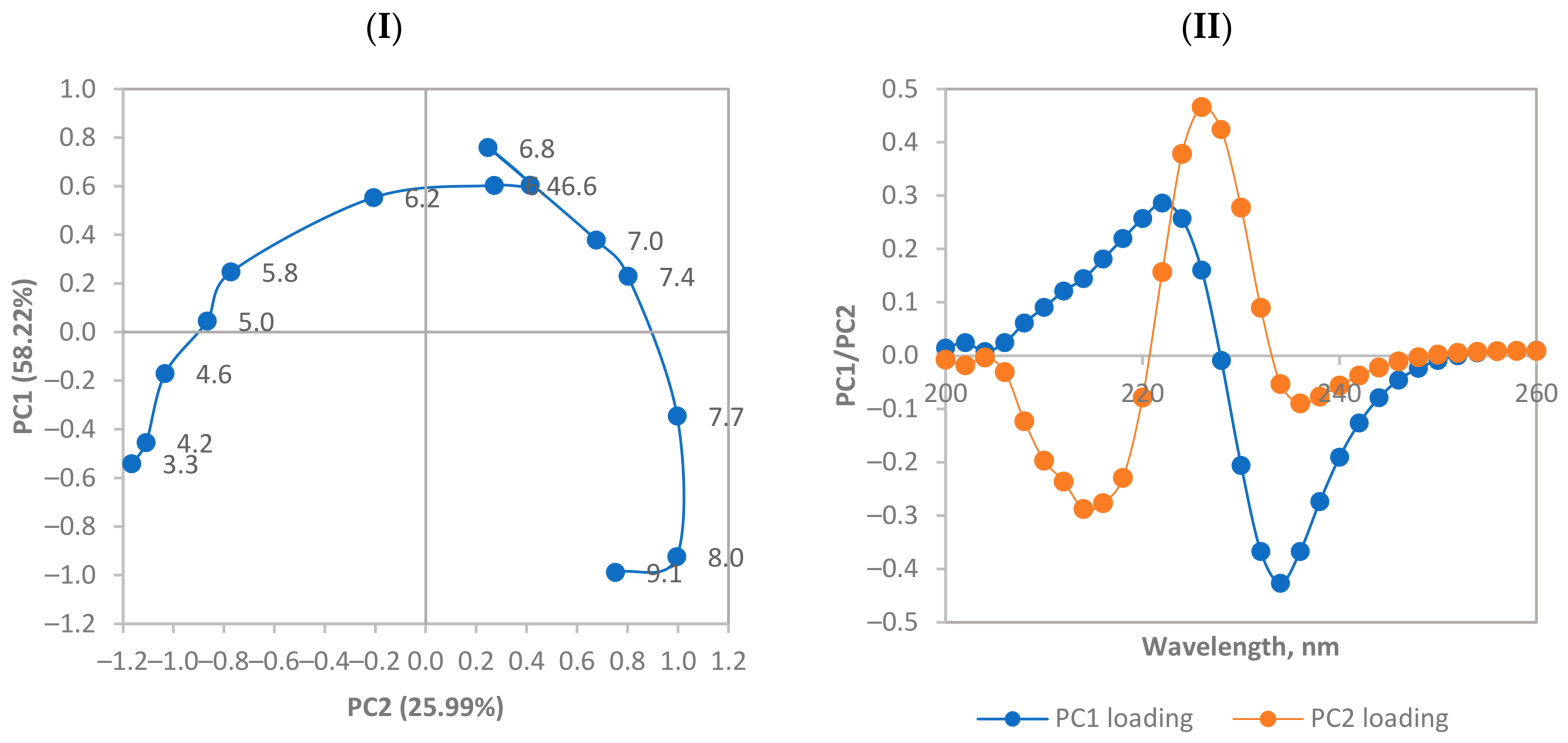
Principal component analysis of the pH-dependent analytical spectral response of cyclosporin A. (**I**) PCA score plot showing the distribution of spectra according to pH in the PC1–PC2 space. (**II**) Loading plots illustrating the wavelength regions contributing to the observed multivariate spectral variability.

Principal component analysis (PCA), mean-centered UV spectra showed that the first two principal components explained 58.22% and 25.99% of the total variance, respectively, corresponding to 84.20% cumulative variance. The score plot revealed an ordered distribution of spectra according to pH in the PC1–PC2 space (Figure 3I). Spectra corresponding to successive pH values occupied systematically changing positions, demonstrating continuous variation in the analytical spectral response of CyA across the investigated buffered media.

The loading profiles showed that both principal components were associated with broad wavelength regions rather than isolated spectral features. The observed multivariate variability therefore involved coordinated changes across the absorption envelope (Figure 3II).

PERMANOVA showed significant multivariate differences among the investigated pH groups (pseudo-F = 652.98, R^2^ = 0.9967, *p* = 0.0001). PERMDISP also indicated significant differences in within-group multivariate dispersion (F = 27.43, *p* = 0.0001).

### 3.3. Spectral Variability Within the Near-Neutral pH Range Relevant to Ophthalmic Formulations and Release Media

To evaluate the analytical relevance of the observed spectral variability under conditions representative of ophthalmic formulations and in vitro release media, the near-neutral part of the pH series was analyzed separately. The investigated pH values (6.60, 6.75, 6.99, 7.37, 7.70, and 7.99) cover a range relevant to ophthalmic formulations, artificial lacrimal media, and in vitro drug-release studies, including the pH interval reported for a commercially available cyclosporine ophthalmic emulsion [14].

Within this relatively narrow pH interval, CyA exhibited measurable spectral variability (Table 4). The apparent absorption maximum shifted from 226 nm at pH 6.60–6.75 to 216 nm at pH 7.99, corresponding to a 10 nm hypsochromic shift. Absorbance at the apparent absorption maximum also varied across the investigated interval, ranging from 0.692 ± 0.003 to 0.751 ± 0.004 AU.

Statistical analysis confirmed significant differences among the investigated buffered media for all evaluated spectral descriptors (Table 3). For the continuous descriptors analyzed by ANOVA, effect sizes were large (η^2^ = 0.873–0.9997), demonstrating substantial variation in the analytical spectral response of CyA even within this formulation-relevant pH interval. The apparent $\lambda_{max}$ also differed significantly among the near-neutral pH conditions (Kruskal–Wallis, *p* = 0.0045, ε^2^ = 1.000).

Using pH 7.37 as the reference condition, the relative change in absorbance at the apparent absorption maximum ranged from +2.6% to −5.5% (Table 4).

## 4. Discussion

### 4.1. Structural Interpretation of the pH-Dependent Analytical Spectral Response

The present study demonstrates that the analytical UV response of cyclosporin A is highly sensitive to the physicochemical properties of the surrounding buffered medium. This conclusion is supported by the progressive hypsochromic shift in the apparent absorption maximum, coordinated changes across the absorption envelope, large effect sizes for all evaluated spectral descriptors, and the ordered distribution of spectra revealed by PCA. These observations indicate that the UV spectral response of CyA cannot be regarded as spectroscopically invariant across the investigated buffered conditions.

**Figure 4:**
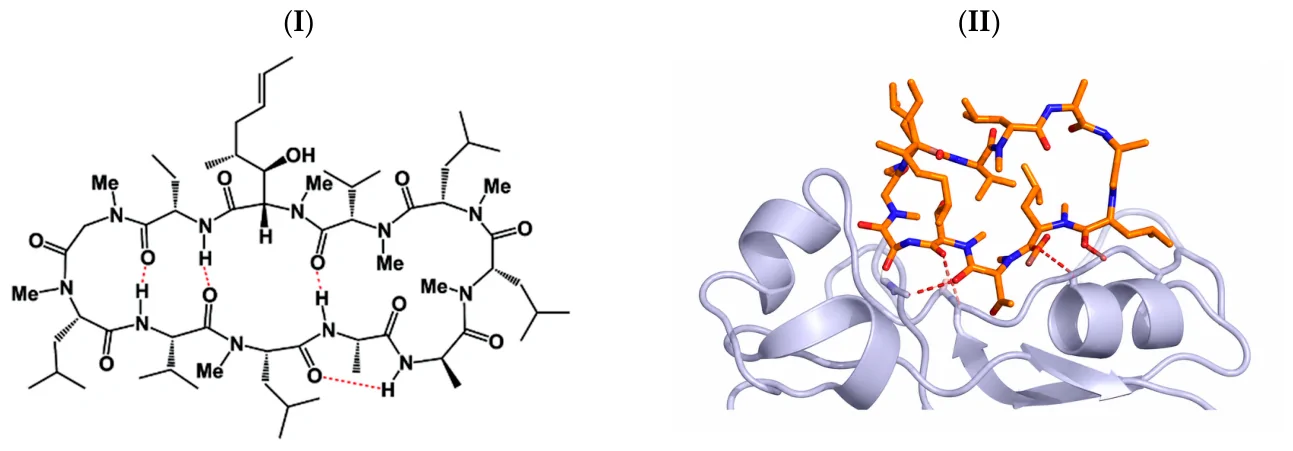
Schematic illustration of structural features associated with the conformational plasticity of cyclosporin A based on published structural studies [7,8,9,10,11]. (**I**) Two-dimensional chemical structure of cyclosporin A with the network of intramolecular hydrogen bonds that contributes to its conformational flexibility. (**II**) Crystal structure of cyclosporin A in complex with protein, demonstrating the conformational rearrangement of the molecule upon protein binding.

The observed spectral behavior can be interpreted in the context of the well-established conformational plasticity of cyclosporins (Figure 4). CyA is recognized as a highly flexible cyclic undecapeptide that exists as an ensemble of rapidly interconverting conformations stabilized by intramolecular hydrogen bonds (IMHBs). Rather than representing a single rigid molecular structure, CyA redistributes between conformational states depending on its physicochemical environment, including solvent polarity, hydration and intermolecular interactions [7,8,9,10].

Among the available structural studies, the work of Hyung et al. [7] provides particularly relevant evidence that cyclosporins can retain distinct IMHB-stabilized conformational families that are experimentally distinguishable. Complementary studies by Lee et al. [9] demonstrated that relatively subtle modifications of the cyclosporin scaffold can alter its chameleonic behavior through changes in intramolecular hydrogen-bond organization, whereas the comprehensive analysis by Corbett et al. [10] highlights the importance of conformational adaptability for the physicochemical behavior and permeability of this class of compounds. More recently, Mácha et al. [11] further illustrated the sensitivity of CyA-derived species to changes in their stabilization environment.

These studies provide a structurally plausible framework for interpreting the spectral behavior observed in the present work. The progressive changes in the UV absorption profile are consistent with redistribution of conformational populations accompanied by modification of the local electronic environment of the peptide chromophores. Accordingly, the measured UV spectrum may represent an ensemble-averaged analytical response arising from multiple coexisting molecular states rather than from a single invariant conformation. PCA and Gaussian deconvolution independently showed that the spectral variability involved coordinated modification of the absorption envelope rather than an isolated change at a single wavelength. These analyses do not directly identify individual conformers but are consistent with the environment-dependent spectral response described here.

### 4.2. Implications for UV-Based Quantification of Cyclosporin A

**Figure 5:**
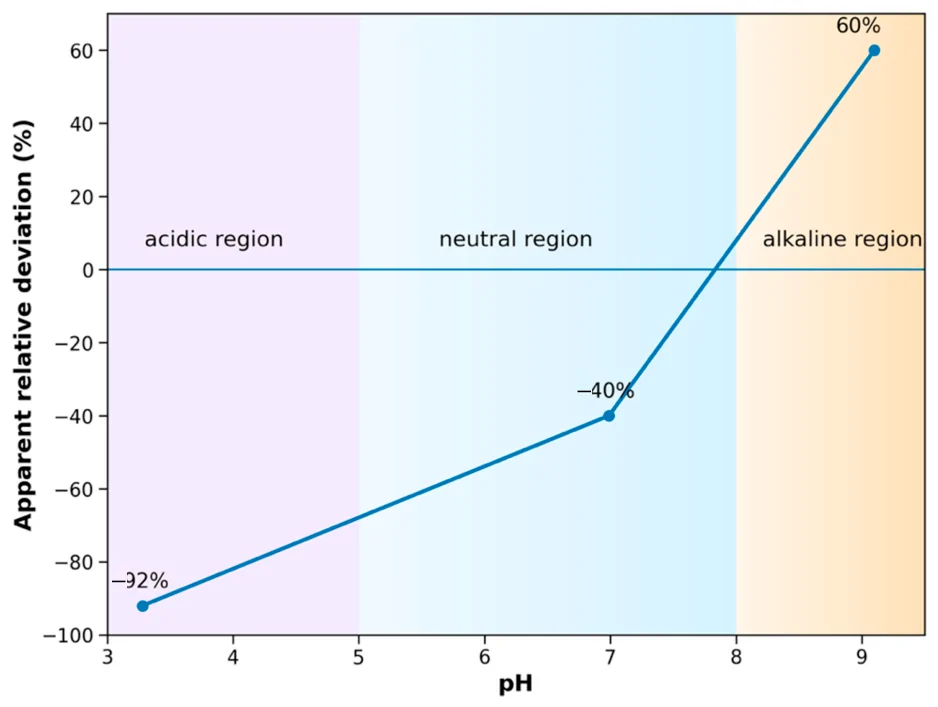
Apparent wavelength-dependent deviation in UV-based quantification of cyclosporin A as a function of pH in citrate–phosphate buffered media.

The analytical consequences of the observed spectral variability extend beyond the displacement of the apparent absorption maximum. Quantitative analysis demonstrated statistically significant differences across the investigated buffered media for all evaluated spectral descriptors, with consistently large effect sizes. Significant spectral differences were also evident within the near-neutral pH interval relevant to ophthalmic formulations and in vitro release media (Figure 5).

These findings indicate that the analytical UV response of CyA depends on the physicochemical environment in which the measurement is performed. Consequently, absorbance measured at a fixed wavelength cannot be assumed to reflect analyte concentration alone when calibration standards and analytical samples differ in buffered medium composition.

This consideration is particularly relevant to UV-based quantification during formulation development, drug-loading experiments, and in vitro release studies. In such applications, calibration standards may be prepared in a medium different from that of the analytical samples, which may consist of buffered release media, artificial lacrimal fluid, or polymer extracts. Under these conditions, differences in analytical spectral response may introduce systematic bias even when the CyA concentration remains unchanged.

The magnitude of the observed spectral variability further emphasizes its analytical relevance. Apparent wavelength-dependent deviations approached 90% under the investigated experimental conditions (Figure 5), whereas measurable differences were also observed within the formulation-relevant near-neutral pH range. Representative changes in apparent $\lambda_{max}$, absorbance, and band position are summarized in Table 5. These findings support the use of matrix-matched calibration whenever possible and careful control of experimental conditions during UV quantification. Where matrix matching cannot be achieved, complementary analytical techniques may provide additional confidence in the quantitative results.

### 4.3. Pharmaceutical Implications for Ocular Drug-Delivery Studies

The analytical observations reported here are directly relevant to the development and evaluation of ocular drug-delivery systems containing cyclosporin A. UV spectroscopy is commonly used to determine drug loading, release kinetics, and residual drug content because of its simplicity, accessibility, and relatively low operational cost [2,3]. The present results demonstrate that the analytical response of CyA depends on the physicochemical characteristics of the surrounding buffered medium and should therefore be considered when quantitative comparisons are made between different experimental systems.

This consideration is particularly important for hydrogel- and contact lens-based delivery platforms, where CyA may be quantified in buffered release media, artificial lacrimal fluid or polymer extracts. Although these media generally operate within a relatively narrow near-neutral pH range, the present study demonstrates that statistically significant spectral variability persists under these formulation-relevant conditions. Consequently, calibration performed in one analytical medium may not be directly transferable to measurements carried out in another medium differing in buffering composition or physicochemical environment.

From a pharmaceutical perspective, part of the variability attributed to differences in loading efficiency or release kinetics may originate from changes in the analytical response itself. Matrix-matched calibration and appropriate selection of calibration conditions may therefore improve the reliability and comparability of quantitative studies involving different formulations, biomaterials, and release protocols.

## 5. Conclusions

The present study demonstrates that the analytical UV spectral response of cyclosporin A is strongly influenced by the surrounding buffered aqueous environment. Progressive changes in the apparent absorption maximum, absorption envelope and absorbance intensity were consistently observed across the investigated pH range and remained detectable within the near-neutral conditions relevant to ophthalmic formulations and in vitro drug-release studies.

Comprehensive univariate and multivariate statistical analyses confirmed that these spectral changes are systematic rather than incidental. The combined evidence obtained from effect size analysis, principal component analysis and spectral deconvolution indicates that the observed variability involves coordinated modification of the entire UV absorption profile rather than isolated changes at a single wavelength.

From an analytical perspective, these findings demonstrate that UV-based quantification of cyclosporin A cannot assume spectroscopic invariance across different buffered media. Consequently, calibration conditions should be carefully matched to the analytical matrix, particularly in pharmaceutical studies involving drug loading, release experiments and biomaterial-based ocular drug-delivery systems.

The present work establishes an analytical framework for the quantitative determination of cyclosporin A in buffered aqueous media. Recognition of the environment-dependent nature of its UV response provides a practical basis for improving the robustness, reliability and comparability of analytical protocols used in pharmaceutical formulation and ocular drug-delivery research.

## Figures and Tables

**Table 1:** Composition of citrate–phosphate buffer solutions.

pH	0.1 M Na_2_HPO_4_ (mL)	0.1 M Citric Acid (mL)
3.28	5.0	20.0
4.19	6.0	19.0
4.55	8.0	16.0
5.00	10.5	14.5
5.75	11.5	13.5
6.21	13.5	11.5
6.44	15.5	9.5
6.60	16.5	8.5
6.75	17.5	7.5
6.99	18.5	6.5
7.37	19.5	5.5
7.70	21.5	3.5
7.99	23.0	2.0
9.10	24.0	1.0

**Table 2:** Experimental conditions for UV–Vis measurements.

Parameter	Condition
Analyte	Cyclosporin A
Concentration	20 µg mL^−1^
Molar concentration	~1.7 × 10^−5^ mol L^−1^
Solvent/matrix	Citrate–phosphate buffer prepared in ultrapure water
pH range	3.28–9.10
Spectral range	200–300 nm
Path length	1 cm
Temperature	Ambient

**Table 3:** Statistical comparison of cyclosporin A spectral descriptors across the investigated buffered media.

Spectral Descriptor	Primary Test	Test Statistic	*p* Value	Effect Size
**Full pH range (3.28–9.10)**				
Apparent $\lambda_{max}$	Kruskal–Wallis	H = 41.00	<0.001	ε^2^ = 1.0000
$A_{\lambda_{max}}$	One-way ANOVA	F(13,28) = 845.86	<0.001	η^2^ = 0.9970
$A_{206}$	One-way ANOVA	F(13,28) = 37.00	<0.001	η^2^ = 0.9450
$A_{224}$	One-way ANOVA	F(13,28) = 1013.74	<0.001	η^2^ = 0.9980
$A_{234}$	One-way ANOVA	F(13,28) = 15585.05	<0.001	η^2^ = 0.9999
Spectral area (200–260 nm)	One-way ANOVA	F(13,28) = 37.27	<0.001	η^2^ = 0.9450
Spectral centroid	One-way ANOVA	F(13,28) = 540.34	<0.001	η^2^ = 0.9960
**Near-neutral pH range (6.60–7.99)**				
Apparent $\lambda_{max}$	Kruskal–Wallis	H = 17.00	0.0045	ε^2^ = 1.0000
$A_{\lambda_{max}}$	One-way ANOVA	F(5,12) = 264.81	<0.001	η^2^ = 0.9910
$A_{206}$	One-way ANOVA	F(5,12) = 16.47	<0.001	η^2^ = 0.8730
$A_{224}$	One-way ANOVA	F(5,12) = 8940.57	<0.001	η^2^ = 0.9997
$A_{234}$	One-way ANOVA	F(5,12) = 9036.76	<0.001	η^2^ = 0.9997
Spectral area (200–260 nm)	One-way ANOVA	F(5,12) = 310.72	<0.001	η^2^ = 0.9920
Spectral centroid	One-way ANOVA	F(5,12) = 1501.64	<0.001	η^2^ = 0.9980

**Table 4:** Spectral characteristics of cyclosporin A within the near-neutral pH range relevant to ophthalmic formulations and release media.

pH	Apparent $\lambda_{max}$ (nm)	Absorbance at $\lambda_{max}$ (AU, Mean ± SD)	Relative Change in $A_{\lambda_{max}}$ vs. pH 7.37 (%)
6.60	226	0.751 ± 0.004	+2.6
6.75	226	0.724 ± 0.001	−1.1
6.99	224	0.707 ± 0.007	−3.4
7.37	222	0.732 ± 0.0004	0.0
7.70	220	0.726 ± 0.003	−0.8
7.99	216	0.692 ± 0.003	−5.5

**Table 5:** Representative analytical consequences of the pH-dependent spectral variability of cyclosporin A.

pH	Apparent $\lambda_{max}$ (nm)	Absorbance at Apparent $\lambda_{max}$ (AU)	$\lambda_{max}$ Shift Relative to pH 3.28 (nm)
3.28	234	~0.96	—
6.99	222–224	~0.71	10–12
9.10	206–212	~0.58	23–28

## Data Availability

The data supporting the findings of this study are available from the corresponding author upon reasonable request.

## References

[1] Rykowska I., Nowak I., Nowak R. (2021). Soft Contact Lenses as Drug Delivery Systems: A Review. Molecules.

[2] Rykowska I., Michałkiewicz O., Nowak I., Nowak R. (2024). Drug-Modified Contact Lenses—Properties, Release Kinetics, and Stability of Active Substances with Particular Emphasis on Cyclosporine A: A Review. Molecules.

[3] Michałkiewicz O., Nowak I., Nowak R., Rykowska I. (2023). Daily Disposable Contact Lenses as a Platform for Ocular Drug Delivery of Cyclosporine A. Physicochem. Probl. Miner. Process..

[4] Yang H., Zhao M., Xing D., Zhang J., Fang T., Zhang F., Nie Z., Liu Y., Yang L., Li J. (2023). Contact lens as an emerging platform for ophthalmic drug delivery: A systematic review. Asian J. Pharm. Sci..

[5] Franco P., De Marco I. (2021). Contact Lenses as Ophthalmic Drug Delivery Systems: A Review. Polymers.

[6] Rykowska I., Nowak I., Nowak R., Michałkiewicz O. (2025). Biodegradable Contact Lenses for Targeted Ocular Drug Delivery: Recent Advances, Clinical Applications, and Translational Perspectives. Molecules.

[7] Hyung S.J., Feng X., Che Y., Stroh J.G., Shapiro M. (2014). Detection of conformation types of cyclosporin retaining intramolecular hydrogen bonds by mass spectrometry. Anal. Bioanal. Chem..

[8] Ono S., Naylor M.R., Townsend C.E., Okumura C., Okada O., Lee H.W., Lokey R.S. (2021). Cyclosporin A: Conformational Complexity and Chameleonicity. J. Chem. Inf. Model..

[9] Lee D., Lee S., Choi J., Song Y.K., Kim M.J., Shin D.S., Bae M.A., Kim Y.C., Park C.J., Lee K.R. (2021). Interplay among Conformation, Intramolecular Hydrogen Bonds, and Chameleonicity in the Membrane Permeability and Cyclophilin A Binding of Macrocyclic Peptide Cyclosporin O Derivatives. J. Med. Chem..

[10] Corbett K.M., Ford L., Warren D.B., Pouton C.W., Chalmers D.K. (2021). Cyclosporin Structure and Permeability: From A to Z and Beyond. J. Med. Chem..

[11] Mácha H., Zápal J., Kuzma M., Luptáková D., Lemr K., Havlíček V. (2024). Exploring the Effects of Cyclosporin A to Isocyclosporin A Rearrangement on Ion Mobility Separation. Anal. Chem..

[12] Oladepo S.A., Xiong K., Hong Z., Asher S.A., Handen J., Lednev I.K. (2012). UV Resonance Raman Investigations of Peptide and Protein Structure and Dynamics. Chem. Rev..

[13] Zsila F. (2022). An overlooked UV spectroscopic tool for sensing coil-to-helix and helix-to-coil conformational transitions of proteins and peptides. Anal. Biochem..

[14] U.S. National Library of Medicine DailyMed: RESTASIS—Cyclosporine Ophthalmic Emulsion. https://dailymed.nlm.nih.gov/dailymed/drugInfo.cfm?setid=8227ca35-77c1-4a08-87a3-7dcefef39bc5.

[15] Bro R., Smilde A.K. (2014). Principal component analysis. Anal. Methods.

